# Physiological Effects of Dietary Protein and Its Optimal Requirement for Early Juvenile Chinese Tapertail Anchovy (*Coilia nasus*)

**DOI:** 10.3390/ani16121887

**Published:** 2026-06-18

**Authors:** Leimin Zhang, Xiaoru Chen, Dongyu Huang, Qunlan Zhou, Xiaodi Xu, Mingchun Ren, Lu Zhang, Hualiang Liang

**Affiliations:** 1 Wuxi Fisheries College, Nanjing Agricultural University, Wuxi 214081, China; 2 Tongwei Agricultural Development Co., Ltd., Key Laboratory of Aquatic Nutrition and Smart Farming, Ministry of Agriculture and Rural Affairs, Aquatic Health and Intelligent Aquaculture Key Laboratory of Sichuan Province, Chengdu 610093, China; 3 Key Laboratory of Integrated Rice-Fish Farming Ecology, Ministry of Agriculture and Rural Affairs, Freshwater Fisheries Research Center, Chinese Academy of Fishery Sciences, Wuxi 214081, China

**Keywords:** *Coilia nasus*, protein, growth, immuno-antioxidant, endoplasmic reticulum stress, apoptosis

## Abstract

To determine the optimal dietary protein level for early juvenile Chinese tapertail anchovy (*Coilia nasus*, initial body weight: 0.87 ± 0.01 g), five feeds containing graded protein levels (35.42%, 39.16%, 42.96%, 46.83%, and 50.65%) were used in an eight-week feeding experiment. Results showed that the growth performance and health status of *C. nasus* were significantly affected by dietary protein. Compared with the 35.42% group, the 42.96% and 46.83% groups’ WGR and SGR are notably higher, and FCR is notably lower. The 46.83% group showed higher crude protein and lower crude lipid contents. The activities of CAT and SOD, and the level of T-AOC, were significantly enhanced in the 42.96% and 46.83% groups. The 42.96–50.65% groups showed significantly higher GPx activities and lower MDA levels, and GR activity was increased in the 46.83% and 50.65% groups. In addition, the 39.16–50.65% groups upregulated the expressions of *il-1β* and *tgf-β* and downregulated *tnf-α*. For endoplasmic reticulum stress and apoptosis, the 42.96–50.65% groups significantly downregulated the expressions of *atf4*, *chop*, and *apaf1*, and the 39.16–50.65% groups upregulated *cflar*, *bcl-2*, and *tradd*, downregulated *bax*, *casp9*, and *casp3*. Quadratic regression analysis using WGR and FCR as indices showed that the optimal protein levels in feed for early juvenile *C. nasus* were 44.31% and 46.56%, respectively.

## Simple Summary

Chinese tapertail anchovy (*Coilia nasus*) is an economically important fish species with growing aquaculture potential. However, information on its dietary protein requirement during the early juvenile stage is lacking, limiting the development of formulated feeds. This study evaluated the effects of different dietary protein levels on growth and physiological health in early juvenile *C. nasus*. The results showed that both insufficient and excessive protein levels impaired fish performance, whereas an appropriate protein level promoted growth and health. The optimal dietary protein requirement was estimated to be 44.31–46.56% of the feed. These findings provide a scientific basis for feed formulation and sustainable aquaculture of *C. nasus*.

## 1. Introduction

Chinese tapertail anchovy (*Coilia nasus*) is of the order Clupeiformes, family Engraulidae, and genus *Coilia*. It is an anadromous fish widely distributed in the Northwest Pacific, including the coastal waters of China, Japan, and South Korea [1]. Owing to its commercial value and consumer acceptance, *C. nasus* has long been an important fishery resource in the Yangtze River basin [2]. Previous studies have reported that its muscle contains considerable amounts of protein and lipid and provides a source of several nutritionally important amino acids [3]. Historically, *C. nasus* was abundant in the Yangtze River; however, since the 1970s, overfishing and habitat degradation have led to a dramatic decline in its population, prompting its classification as Endangered (EN) on the International Union for Conservation of Nature (IUCN) Red List of threatened species [4]. Notably, a study indicated that farmed *C. nasus* exhibits comparable nutritional quality to its wild counterparts and can serve as a viable substitute [5]. Therefore, advancing the aquaculture of *C. nasus* could not only help meet its market demand but also support the conservation and recovery of wild populations [2,4].

Traditional *C. nasus* culture relies on live bait, which offers benefits in terms of feed attraction and survival, but also presents prominent disadvantages, including high cost and risk, such as the presence of pathogens and water contamination [5]. In contrast, artificial feed can reduce the cost and risk while maintaining feed attraction and survival. Artificial feed farming relies on nutritionally balanced feed, and different aquatic species have distinct nutritional requirements [6], so clarifying the precise requirements of nutrients in feed for *C. nasus* is essential for promoting its aquaculture industry. Thus, quantifying nutrient requirements through nutritional science is a prerequisite for designing optimal feeds and represents a primary task in aquaculture.

Currently, the dietary nutrient requirements of *C. nasus* remain largely unknown, which has constrained the development of its efficient artificial culture. Protein is an essential nutrient that serves as a structural component of tissues and plays a vital role in growth, survival, and physiological regulation in fish [7,8]. Therefore, determining the optimal dietary protein requirement is a prerequisite for developing nutritionally balanced formulated feeds for *C. nasus*. Previous studies have demonstrated that fish growth generally increases with dietary protein level up to an optimal range, beyond which growth performance may plateau or even decline [9,10,11,12]. In addition to growth, dietary protein significantly affects body composition and protein deposition, with appropriate protein levels promoting protein accumulation in fish tissues [13,14]. Furthermore, protein is closely associated with immune function and antioxidant capacity, and optimal dietary protein levels have been shown to enhance antioxidant defenses and immune responses in several fish species [15,16]. Therefore, establishing the dietary protein requirement of *C. nasus* is essential for improving growth performance and maintaining physiological health.

Based on the aforementioned background, this study conducted a feeding trial with formulated feeds containing graded protein levels for early juvenile *C. nasus*, with the objectives of quantitatively determining its optimal dietary protein requirement and systematically investigating the effects of protein level on growth performance, body composition, immune-antioxidant capacity, and other physiological parameters. The findings are expected to provide essential data support for developing species-specific formulated feeds, thereby promoting the advancement of *C. nasus* aquaculture and supporting the recovery of its depleted wild populations.

## 2. Materials and Methods

### 2.1. Diet Preparation

Five isolipidic/isoenergetic experimental feeds were formulated using fish meal, Antarctic krill (*Euphausia superba*) meal, and wheat flour as the main protein sources, and fish oil as the main lipid source. The protein content of each feed was adjusted by varying the proportions of fish meal and wheat flour. The inclusion level of monocalcium phosphate was adjusted according to the fish meal and wheat flour levels, which balanced the phosphorus levels in each group of feed formulas to maintain consistency. The composition and nutrient content of the feeds are presented in Table 1. Feed production comprised crushing, sieving, weighing, mixing, and pelleting [17]. After crushing and 80-mesh sieving, ingredients were mixed with water and oil. An extruder of model TSE65 from Beijing Modern Yanggong Machinery S and T Development Co., Ltd. (Beijing, China) was used to process the mixture into 1 mm diameter pellet feed. The feed was naturally dried in a cool, shaded place and stored at −20 °C. Samples from each feed were randomly taken for nutrient analysis. The protein content of the five feeds was 35.42%, 39.16%, 42.96%, 46.83%, and 50.65%, respectively.

### 2.2. Experimental Procedure

**Figure 1:**
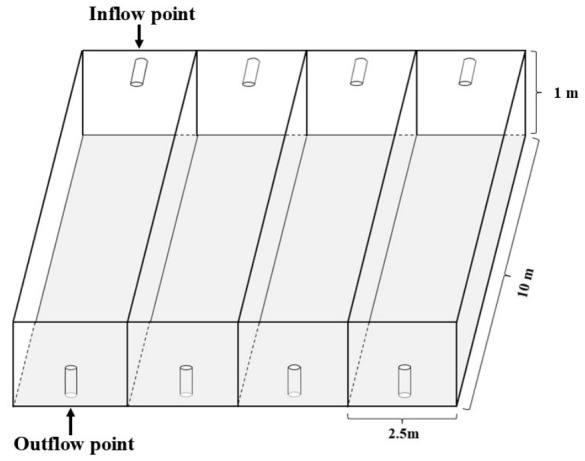
Schematic diagram of the structure of a cement pool for aquaculture.

To comprehensively investigate the impacts of dietary protein level on *C. nasus*, an eight-week feeding trial was designed. The experiment was conducted in a greenhouse at the Yangzhong Base of the Freshwater Fisheries Research Center (FFRC), Chinese Academy of Fishery Sciences (Yangzhong, China). The culture pools were rectangular cement pools measuring 10 m × 2.5 m × 1 m (L × W × H) (Figure 1), and the water height was 0.4 m. Each pool was maintained under static water conditions, equipped with an inlet and an outlet, and one-third of the water was renewed daily. Shade nets were placed above the cement pools to prevent *C. nasus* from suffering stress caused by sunlight exposure.

The experimental fish were provided by Zhenjiang Jiangzhiyuan Technology Co., Ltd. (Zhenjiang, China). A total of 6000 early juvenile *C. nasus* with robust health and uniform size (initial body weight of 0.87 ± 0.01 g). According to the developmental stages commonly used in *C. nasus* aquaculture, individuals of this weight are classified as early juveniles were selected and divided into five groups (three replicates each), totaling 15 pools. During the acclimation period, commercial feed was used, and dead fish were promptly replaced to maintain a consistent stocking density. After a two-week acclimation period, the formal experiment began using the experimental feed, and dead fish were no longer replaced. Throughout the culture period, fish were fed three times daily at 7:30, 12:30, and 17:30 according to apparent satiation, and uneaten feed was collected from the bottom of the pool 30 min after each feeding ceased, and the weight was recorded to calculate the feed conversion ratio (FCR). The mortality of fish was recorded daily.

The physicochemical parameters of the culture water were measured using reagent kits provided by Sampsistemi Biochemistry and Technology Co., Ltd. (Beijing, China). Among these parameters, water temperature, dissolved oxygen, and pH were measured daily, while ammonia nitrogen and nitrite nitrogen were measured once every three days. The water temperature was maintained at 28 ± 1 °C, dissolved oxygen was 6.2 ± 0.6 mg/L, ammonia nitrogen concentration was below 0.1 mg/L, nitrite nitrogen concentration was below 0.05 mg/L, and the pH value was 7.4 ± 0.3 throughout the experiment.

### 2.3. Sample Collection

Following the eight-week feeding trial, fish were fasted for 24 h to empty food residues from the digestive tract. Fish in each cement pool were then collectively weighed and counted to obtain data for growth performance, FCR, and survival rate (SR), etc. Subsequently, 20 fish were randomly sampled from each pool and stored at −20 °C for later whole-body proximate composition analysis. Finally, 3 fish per pool were dissected to collect liver tissue samples, which were then preserved at −80 °C for future determination of enzyme activities and analysis of relevant gene expression levels.

### 2.4. Chemical Analysis

The compositions of the whole fish body and the experimental feeds were determined by methods based on the AOAC standards [18]. Moisture was determined using the drying method, crude protein content using Kjeldahl nitrogen determination, crude lipid content using Soxhlet extraction, and crude ash using the ashing method. Hepatic antioxidant capacity and activities of immune-related enzymes were determined by relevant kits purchased from Nanjing Jiancheng Bioengineering Institute (Nanjing, China). After mixing liver tissue and saline at a ratio of 1 (g): 9 (mL) to make a 10% tissue homogenate, the total protein (TP) content was first determined for the subsequent calculation of the results of other indices. The specific determination methods and instruments used for each of the above indicators are shown in Table 2.

### 2.5. RNA Extraction and RT-qPCR Analysis

The relative expression levels of mRNA of relevant genes in the liver of *C. nasus* were detected using the real-time quantitative PCR (RT-qPCR) method [19]. Table 3 lists the primer sequences used in the experiment, which were obtained from previous studies and synthesized by Sangon Bioengineering Co., Ltd. (Shanghai, China). Based on the relative stability of expression, *β-actin* was selected as the internal reference gene.

The RT-qPCR process was performed as follows: (1) Total RNA from fish liver was isolated with RNA extraction reagent (model R711-01). The RNA was isolated through tissue lysis, centrifugation, precipitation, and resuspension, and was then dissolved in diethyl pyrocarbonate (DEPC)-treated RNase-free water. (2) RNA concentration and quality were assessed using a NanoDrop 2000 microspectrophotometer (Thermo Fisher Scientific, Waltham, MA, USA). The RNA concentration was diluted to 60 ng/μL, with the A260/A280 ratio of 1.8–2.0, ensuring that the RNA purity met the requirements for subsequent assays. The RNA solution was then stored at −80 °C. (3) qRT-PCR was run with the One-Step RT-PCR SYBR Green kit on a CFX96 system (Bio-Rad, Hercules, CA, USA). (4) Target gene mRNA expression was calculated using the 2^−ΔΔCt^ method. The reagents mentioned in this section were purchased from Vazyme Biotech Co., Ltd. (Nanjing, China).

### 2.6. Statistical Analysis

Before statistical analysis, data normality was assessed by the Shapiro–Wilk test, and variance homogeneity by Levene’s test. Data meeting the assumptions of normality and homogeneity of variances were analyzed using SPSS 25.0. Group comparisons used one-way ANOVA, and Duncan’s test was used for multiple comparisons. Data are presented as “mean ± standard deviation (SD),” and the significant level was set at *p* < 0.05. To evaluate the protein requirement of *C. nasus* in feed, the data were regressed using weight gain rate (WGR) and FCR as evaluation indicators. The quadratic regression model, which provided a better fit based on higher R^2^ values compared to the linear model, was selected.

## 3. Results

### 3.1. Growth Performance and Whole Fish Body Composition

**Figure 2:**
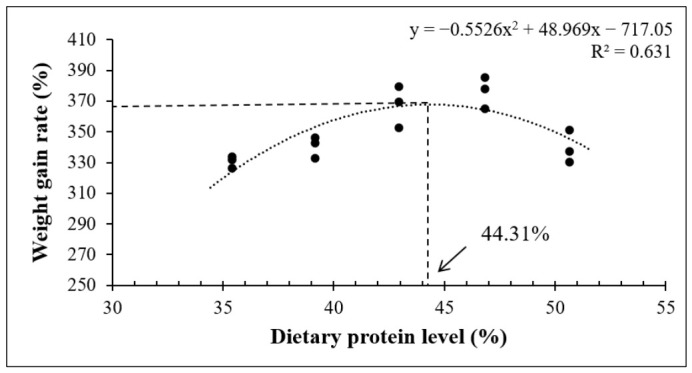
Quadratic regression analysis of WGR with different dietary protein levels. The dotted line represents the quadratic regression curve, and the dashed line indicates the x- and y-coordinates corresponding to the maximum value on the curve.

**Figure 3:**
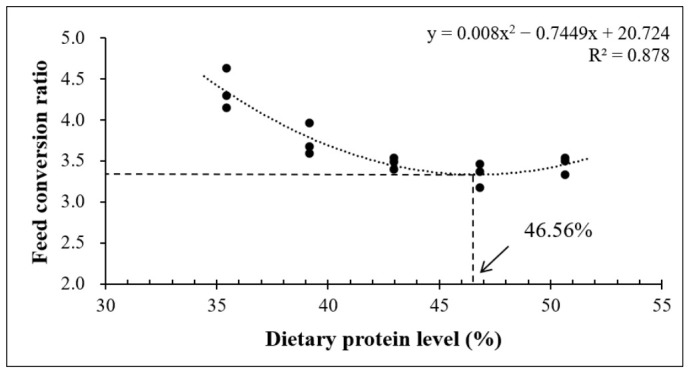
Quadratic regression analysis of FCR with different dietary protein levels. The dotted line represents the quadratic regression curve, and the dashed line indicates the x- and y-coordinates corresponding to the minimum value on the curve.

As shown in Table 4, the dietary protein level had a significant effect on the growth performance of *C. nasus*. Compared to the 35.42% group, the 46.83% protein level significantly increased the final body weight (FBW) (*p* < 0.05). In the 42.96% and 46.83% groups, WGR and specific growth rate (SGR) were significantly higher than those of other groups (*p* < 0.05), while FCR was significantly lower (*p* < 0.05). The SR of *C. nasus* tended to increase and then stabilize, but differences were not significant (*p* > 0.05). The results of quadratic regression analysis using WGR and FCR as indices showed that the optimal protein levels in feed for early juvenile *C. nasus* were 44.31% (Figure 2) and 46.56% (Figure 3), respectively.

Table 5 shows the effect of dietary protein level on the body composition of early juvenile *C. nasus*. With the protein level increased, the crude protein content was significantly higher in the 42.96–50.65% groups than in the 35.42% group (*p* < 0.05), while the crude lipid content was significantly lowest in the 46.83% group (*p* < 0.05). Moisture and ash contents were not significantly affected by dietary protein level (*p* > 0.05).

### 3.2. Hepatic Antioxidant Capacity and Immunity

**Figure 4:**
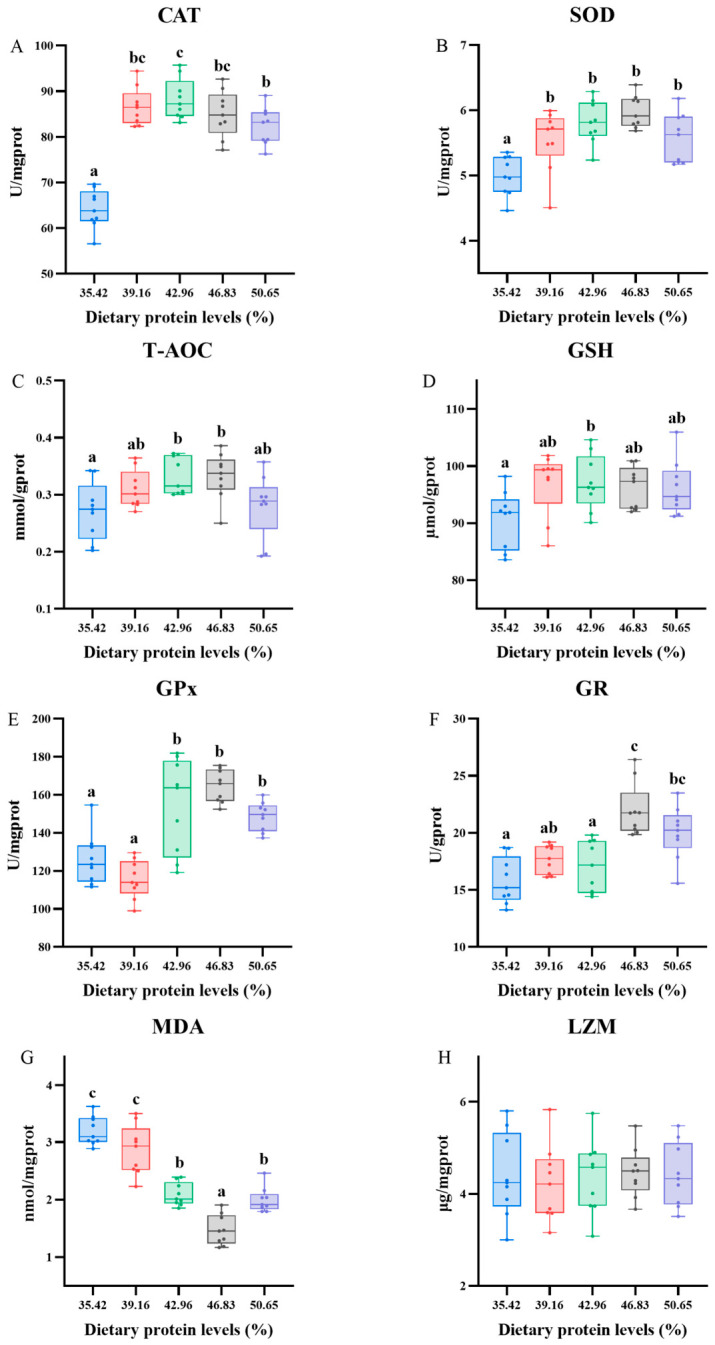
Effects of dietary protein levels on immuno-antioxidant capacity of *C. nasus*. Data are presented as mean value with SD (*n* = 3). Different superscripted letters on the same row indicated a significant difference (*p* < 0.05). (**A**) CAT: Catalase; (**B**) SOD: Superoxide dismutase; (**C**) T-AOC: Total antioxidant capacity; (**D**) GSH: Glutathione; (**E**) GPx: Glutathione peroxidase; (**F**) GR: Glutathione reductase; (**G**) MDA: Malondialdehyde; (**H**) LZM: Lysozyme.

In terms of antioxidant capacity and immunity, the results presented in Figure 4 suggest that compared to the 35.42% group, there was a significant increase in the activities of catalase (CAT) and superoxide dismutase (SOD) in the 39.16–50.65% groups (*p* < 0.05). The 42.96–50.65% groups exhibited a considerable rise in glutathione peroxidase (GPx) activity and a corresponding decrease in malondialdehyde (MDA) content (*p* < 0.05). The total antioxidant capacity (T-AOC) was found to be substantially elevated in the 42.96% and 46.83% groups (*p* < 0.05). Furthermore, the 42.96% group had the highest content of glutathione (GSH) (*p* < 0.05). The activity of glutathione reductase (GR) was considerably increased in the 46.83% and 50.65% groups (*p* < 0.05). The protein level did not significantly impact the LZM activity (*p* > 0.05).

**Figure 5:**
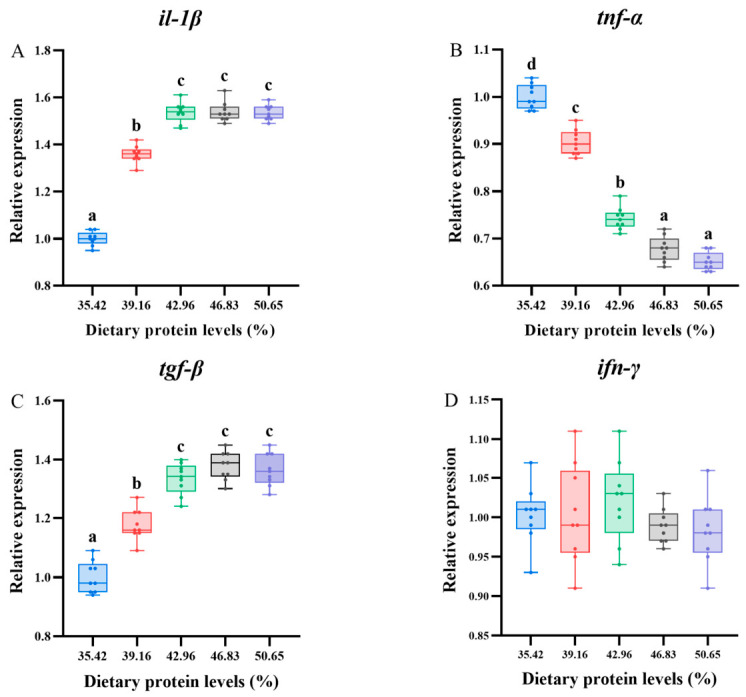
Relative expression of immuno-genes mRNA in *C. nasus* liver. Data are presented as mean value with SD (*n* = 3). Different letter labels indicated significant differences (*p* < 0.05). (**A**) *il-1β*: Interleukin-1 beta; (**B**) *tnf-α*: Tumor necrosis factor-alpha; (**C**) *tgf-β*: Transforming growth factor-beta; (**D**) *ifn-γ*: Interferon-gamma.

At the molecular level, Figure 5 illustrates the expression of mRNA of immune-related genes. Compared to the 35.42% group, the 39.16–50.65% groups significantly upregulated the expressions of *il-1β* and *tgf-β*, while significantly downregulating the expression of *tnf-α* (*p* < 0.05). The expression of *ifn-γ* was not significantly affected by the protein level (*p* > 0.05).

### 3.3. The mRNA Expression Levels of Endoplasmic Reticulum Stress (ERS) and Apoptosis-Related Genes

**Figure 6:**
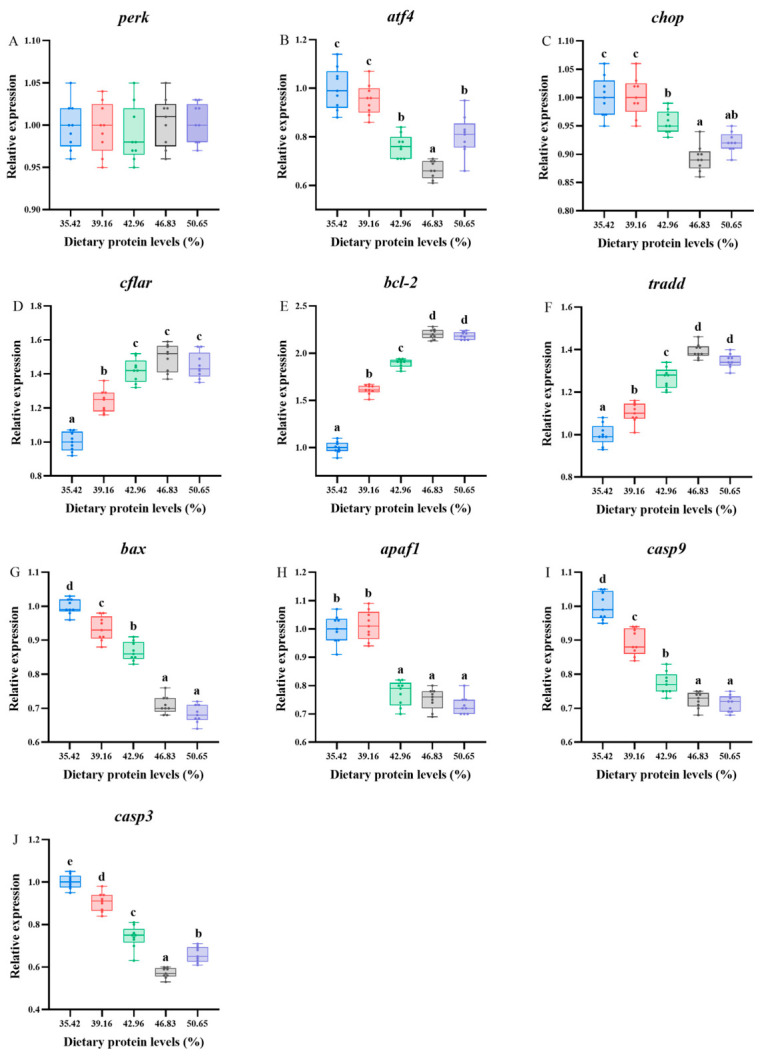
Relative expression of ERS and apoptosis-related genes mRNA in *C. nasus* liver. Data are presented as mean value with SD (*n* = 3). Different letter labels indicated significant differences (*p* < 0.05). (**A**) *perk*: Protein kinase r-like endoplasmic reticulum kinase; (**B**) *atf4*: Activating transcription factor 4; (**C**) *chop*: C/EBP homologous protein; (**D**) *cflar*: CASP8 and FADD-like apoptosis regulator; (**E**) *bcl-2*: B-cell lymphoma 2; (**F**) *tradd*: TNF receptor associated death domain; (**G**) *bax*: BCL2-associated X protein; (**H**) *apaf1*: Apoptotic peptidase activating factor 1; (**I**) *casp9*: Caspase 9; (**J**) *casp3*: Caspase 3.

Regarding the endoplasmic reticulum stress (ERS)-related gene expression, Figure 6 shows that compared to the 35.42% group, the 42.96–50.65% groups significantly downregulated the expressions of *atf4* and *chop* (*p* < 0.05); different protein levels did not significantly affect the expression of *perk* (*p* > 0.05). For anti-apoptosis, the expressions of *cflar* and *bcl-2* were significantly higher in the 39.16–50.65% groups than in the 35.42% group (*p* < 0.05), and both of them had the maximum value in the 46.83% group. For pro-apoptosis, the expression of *tradd* was significantly upregulated, and *bax*, *casp9,* and *casp3* were significantly downregulated in the 39.16–50.65% groups compared to the 35.42% group (*p* < 0.05). The expression of *apaf1* was significantly lower in the 42.96–50.65% groups than in the other groups (*p* < 0.05).

## 4. Discussion

### 4.1. Effect of Dietary Protein on Growth Performance and Fish Body Composition of C. nasus

Protein is essential for the growth, metabolism, and reproduction of fish [22]. Generally, within a certain range, increasing dietary protein levels could promote fish growth [23], while insufficient levels inhibit growth, whereas excessive levels increase metabolic burden, feed waste, and water pollution [24,25]. In this study, no significant differences in SR of *C. nasus* were observed among groups, indicating that survival was unaffected by dietary protein level under the current experimental conditions, consistent with findings in tilapia (*Oreochromis niloticus*) and blunt snout bream (*Megalobrama amblycephala*) [26,27]. With increasing dietary protein level, the FBW, WGR, and SGR of *C. nasus* first increased and then decreased, while the FCR showed an opposite trend. These results align with findings of most related studies, such as grass carp (*Ctenopharyngodon idella*) [10], ide (*Leuciscus idus*) [11], and Asian red-tailed catfish (*Hemibagrus wyckioides*) [28]. The reason for this may be that when dietary protein levels exceed the optimal requirement of fish, the excess protein is deaminated to energy rather than being synthesized. High-protein diets usually contain lower levels of non-protein energy sources, leaving fish to metabolize protein for energy, which significantly reduces protein utilization efficiency [29]. Additionally, excessive protein intake increases ammonia production through deamination, imposing a metabolic burden. Accumulated ammonia is known to be toxic to fish, further impairing growth and feed utilization [30]. Based on WGR and FCR, the optimal dietary protein level for *C. nasus* was determined to be 44.31–46.56%. Within this range, *C. nasus* could achieve maximum growth and optimal feed utilization. Notably, the growth performance and SR of *C. nasus* in this study were lower than those of highly domesticated aquaculture species. This may be attributed to its biological characteristics as a migratory fish with high sensitivity to environmental and handling stress. Furthermore, artificial culture techniques for this species are still at an early stage, and its nutritional requirements and feeding strategies remain largely undefined; thus, the formulated diets used in this study may not have fully met their nutritional needs. Early juveniles are particularly sensitive to diet and environmental changes, which may also have contributed to lower survival.

Moisture, crude protein, lipid, and ash contents reflect fish nutritional status [31]. Studies indicate that protein content in animals maintains a dynamic equilibrium, and dietary protein can influence body protein metabolism. Only when dietary protein reaches an appropriate level does body protein content increase [32]. In this study, both crude protein and lipid contents in *C. nasus* varied with dietary protein level, with the former showing a tendency to increase and then stabilize, while the latter was decreasing and then stabilizing, suggesting that an optimal dietary protein level is favorable to the protein deposition in *C. nasus*, which may be one of the reasons for the growth promotion. Similar conclusions have been reported for American eel (*Anguilla rostrata*) [33], grass carp [10], ide [11], and bagrid catfish (*Pseudobagrus fulvidraco*) [34]. This positive correlation underscores the importance of optimizing dietary protein levels to enhance protein deposition in fish. Regarding the opposite trends between crude protein and crude lipid contents, it has been suggested that low dietary protein may have limited the supply of essential amino acids required for growth and protein synthesis in fish, leading to excess free amino acids being metabolized into body lipids. Conversely, when dietary protein level is appropriately increased, more protein is ingested and absorbed, with surplus amino acids being utilized for protein synthesis and lipid deposition being reduced [35,36].

### 4.2. Effect of Dietary Protein on Immunity and Antioxidant Capacity of C. nasus

Cells produce oxygen free radicals during metabolism, and excess free radicals are the primary cause of oxidative stress and cellular damage [37]. To counteract oxidative damage, cells possess an antioxidant system, including enzymes such as CAT, SOD, GPx, and non-enzymatic substances like GSH, which eliminate reactive oxygen species and mitigate oxidative damage [38]. In this study, as dietary protein level increased from 35.42% to 46.83%, hepatic activities of CAT, SOD, GPx, and GR, T-AOC, and GSH levels in *C. nasus* progressively increased. Concurrently, MDA content gradually declined and was significantly lower in high-protein groups (42.96–50.65%) than in the low-protein groups (35.42% and 39.16%). These dose-dependent trends indicate that moderate increases in dietary protein enhance antioxidant capacity and reduce oxidative damage in *C. nasus*, consistent with findings in farmed tilapia [15] and Asian red-tailed catfish [28]. The enhanced antioxidant capacity is primarily attributed to the fact that antioxidant enzymes are essentially proteins. Elevated protein intake enhances the synthesis of antioxidant enzyme proteins, consequently increasing their contents and activities [39]. In addition to this direct mechanism, some researchers have proposed an alternative explanation for the reduction in MDA content: sufficient protein supply favors amino acid catabolism over fatty acid oxidation for energy production, reducing free radical generation during fatty acid β-oxidation, and consequently decreasing MDA production [39,40]. Thus, the reduction in MDA content with increased dietary protein level may be achieved through a dual regulatory pathway.

Immunity serves as another critical indicator of fish health. A robust immune system not only initiates rapid immune responses but also effectively modulates inflammation to maintain homeostasis [41]. LZM is a crucial non-specific immune factor ubiquitously distributed in fish bodily fluids and tissues, where it compromises the structural integrity of bacteria, viruses, and other pathogens [42]. In this study, hepatic LZM activity in *C. nasus* was unaffected by dietary protein level, consistent with findings in hybrid tilapia (*Oreochromis niloticus × O. aureus*) [43], but contrasting with rainbow trout (*Oncorhynchus mykiss*), where low-protein feed significantly suppressed LZM activity [44]. Such discrepancies may be attributed to species-specific physiological and immunoregulatory mechanisms, reflecting differential immune sensitivity to dietary protein variations across fish species. In addition to enzymatic activities at the protein level, gene expression level can provide deeper insights into molecular regulatory mechanisms [15]. Cytokine-driven inflammatory responses are pivotal for immunity, where a balanced interplay between pro-inflammatory and anti-inflammatory factors maintains immune homeostasis [45]. Although research on dietary protein’s effects on inflammatory factors remains limited, existing studies have shown that optimal dietary protein can modulate inflammatory responses. For instance, it downregulates pro-inflammatory cytokines like *tnf-α* and *il-1β* in tilapia [15], downregulates *tnf-α* and *il-1β* while upregulating the anti-inflammatory factor *tgf-β* in grass carp [46], thereby alleviating inflammation and enhancing immune homeostasis. In this study, high-protein groups (42.96–50.65%) had significantly higher *tgf-β* expression than the low-protein groups (35.42% and 39.16%), and *tnf-α* exhibited the opposite trend. These results align with the aforementioned studies, suggesting that higher dietary protein levels promote immune homeostasis in *C. nasus*. Oxidative stress is a key trigger for *tnf-α* upregulation [15], given that appropriate protein levels mitigate oxidative stress, this may contribute to *tnf-α* suppression. Pro-inflammatory and anti-inflammatory factors typically exhibit antagonistic relationships, but unlike the other studies mentioned above [15,46], hepatic *il-1β* expression in *C. nasus* was significantly increased with increasing dietary protein. In this regard, it has been suggested that under adequate protein nutrition, *il-1β* can be appropriately upregulated to support immune defense when needed, while *tgf-β* is concurrently upregulated to exert negative feedback and prevent excessive inflammation, whereas *tnf-α* is preferentially suppressed due to its potent pro-inflammatory effects and high energy cost [47,48]. Similar observations were reported in grass carp, where *il-1β* and *tgf-β* concurrently increased within a certain protein range while *tnf-α* progressively declined, indicating enhanced immune status [46]. These results indicate that optimal dietary protein enhanced antioxidant capacity, attenuated inflammatory responses, and maintained immune homeostasis in *C. nasus*.

### 4.3. Effect of Dietary Protein on Endoplasmic Reticulum Stress (ERS) and Apoptosis of C. nasus

The endoplasmic reticulum (ER) is the primary site for protein synthesis and maturation. Disruption of ER homeostasis by factors such as hypoxia, nutritional deficiency, or redox imbalance can induce ER stress (ERS) [49], which is a critical factor in tissue structural damage [50]. Previous studies have demonstrated that oxidative stress triggers ERS in mammals [51]. Optimal dietary protein increases glutamate content in grass carp muscle [52], and glutamate can be used to synthesize glutamine, which could alleviate ERS in rats [53]. These findings suggest that optimal dietary protein may be associated with the regulation of ERS-related responses in animal tissues, potentially via the glutamate-glutamine pathway. During ERS, *perk*, one of the ERS sensors, is activated and fulfills a dual role: suppressing ERS by inhibiting protein synthesis, and concurrently activating downstream *atf4*, which upregulates chop expression, leading to *bax* transcription and cell death [54]. In the present study, hepatic *perk* expression in *C. nasus* showed no significant differences among groups, whereas its downstream effectors *atf4* and *chop* were significantly downregulated in the high-protein groups (42.96–50.65%). These results are consistent with observations in grass carp [55], suggesting that optimal dietary protein intake may be associated with improved ER stress-related gene expression profiles and potentially contributes to ER homeostasis regulation. Consistent with the antioxidant discussion above and findings from other studies, a dual protective mechanism is proposed based on gene expression patterns and previous studies. Adequate dietary protein provides substrates for structural protein synthesis, thereby enhancing tissue resilience against damage. Additionally, it improves the ability of fish to cope with ERS by boosting antioxidant capacity [56]. These findings suggest that adequate dietary protein may contribute to cellular stress management through multiple complementary mechanisms.

Apoptosis is a conserved cell death pathway that mediates programmed cell elimination and maintains organismal homeostasis during normal eukaryotic development [57]. However, sustained stimuli such as oxidative stress and ERS can induce excessive apoptosis, leading to severe tissue damage, organ dysfunction, and even systemic failure [58]. Based on functional classification, apoptotic regulators can be categorized into anti-apoptotic proteins (e.g., *cflar* and *bcl2*) and pro-apoptotic proteins (e.g., *bax*, *apaf1*, *casp9*, and *casp3*) [59]. This study showed that increasing dietary protein progressively upregulated hepatic *cflar* and *bcl2*, while downregulating *bax*, *apaf1*, *casp9*, and *casp3* of *C. nasus* in a significant dose-dependent manner. Mechanistically, ERS upregulates *bax* and *apaf1* expressions, leading to *casp9* activation and subsequent *casp3*-mediated apoptosis [60]. Anti-apoptotic *bcl2* suppresses *bax*, while *cflar* inhibits caspases overexpression, thereby maintaining apoptotic balance [59]. Consistent with the expression trends of ERS-related factors discussed above, similar findings from other studies suggest that optimal protein intake sustains ER homeostasis to regulate apoptotic balance [46,55]. Furthermore, Liu proposed that the reduction in pro-apoptotic factor expression by optimal dietary protein level may also involve suppression of the death receptor pathway [54]. *Tradd* is typically recognized as a classical pro-apoptotic factor [59], but in the present study, its expression correlated positively with dietary protein level, contrasting with other pro-apoptotic factors. Regarding this observation, other studies have indicated that under low-intensity stress or cytoprotective conditions, the upregulation of *tradd* may not promote apoptosis but rather indicate activation of survival programs, recruiting TRAF2/RIPK1 to activate the NF-κB pathway and upregulate anti-apoptotic genes, thereby inhibiting excessive apoptosis [61,62]. This further reflects the maintenance of tissue homeostasis. However, the precise regulatory mechanisms in *C. nasus*, or in fish more broadly, require further investigation. These results indicate that appropriate dietary protein content is associated with improved regulation of ER stress-related and apoptosis-related gene expression, suggesting a potential role in maintaining cellular homeostasis in the liver of *C. nasus*.

## 5. Conclusions

This study conducted a feeding trial to assess the optimal protein requirement for *C. nasus*. Key findings revealed that an appropriate dietary protein level not only significantly improved growth performance, feed utilization, and body composition but also enhanced hepatic antioxidant capacity, alleviated inflammation, and maintained ER homeostasis and apoptotic balance. Based on WGR and FCR, the optimal dietary protein level for *C. nasus* ranges from 44.31% to 46.56%. Future research should further investigate the underlying mechanisms of dietary protein effects, quantify protein requirements at different growth stages of *C. nasus*, and establish a database for other essential nutrients.

## Figures and Tables

**Table 1:** Composition and measured nutrient contents of the feeds (% dry matter).

Ingredients	Diets (Protein Level, %)				
	35.42	39.16	42.96	46.83	50.65
Fish meal ^1^	35.00	41.80	48.60	55.40	62.20
Antarctic krill meal ^1^	10.00	10.00	10.00	10.00	10.00
Wheat flour ^1^	35.00	30.00	25.00	20.00	15.00
Fish oil	4.50	4.02	3.54	3.06	2.58
Choline chloride	0.50	0.50	0.50	0.50	0.50
Vitamin C (35%)	0.05	0.05	0.05	0.05	0.05
Monocalcium phosphate	3.00	2.34	1.68	1.02	0.36
Vitamin premix ^2^	1.00	1.00	1.00	1.00	1.00
Mineral premix ^2^	1.00	1.00	1.00	1.00	1.00
Rice bran	5.00	4.30	3.40	2.70	1.80
Microcrystalline cellulose	4.95	4.99	5.23	5.27	5.51
Analyzed proximate composition					
Crude protein	35.42	39.16	42.96	46.83	50.65
Crude lipid	10.52	10.43	10.39	10.38	10.29
Gross energy (KJ/g)	17.22	17.30	17.28	17.33	17.25

Note: ^1^ Fish meal: crude protein 67.8%, crude lipid 9.3%; Antarctic krill meal, crude protein 61.8%, crude lipid 11.6%; wheat flour, crude protein 13.1%, crude lipid 4.0%. All of them were obtained from Wuxi Tongwei Feedstuffs Co., Ltd. (Wuxi, China). ^2^ Vitamin premix and mineral premix were obtained from HANOVE Biotechnology Co., Ltd. (Wuxi, China). The specific components were as follows. Vitamin premix (IU or mg/kg of premix): Vitamin A 900,000 IU; Vitamin D 250,000 IU; Vitamin E 4500 mg; Vitamin K 3220 mg; Vitamin B1 320 mg; Vitamin B2 1090 mg; Vitamin B5 2000 mg; Vitamin B6 5000 mg; Vitamin B12 116 mg; Pantothenate 1000 mg; Folic acid 165 mg; Biotin 50 mg; Niacin acid 2500 mg. Mineral premix (g/kg of premix): calcium phosphate 20 g; sodium chloride 2.6 g; potassium chloride 5 g; magnesium sulphate 2 g; ferrous sulphate 0.9 g; zinc sulphate 0.06 g; cupric sulphate 0.02 g; manganese sulphate 0.03 g; sodium selenate 0.02 g; cobalt chloride 0.05 g; potassium iodide 0.004 g.

**Table 2:** Analysis methods used in the experiment.

Items	Methods	Testing Equipment/Assay Kits
Moisture	Drying method, 105 °C	Electric blast drying oven (Shanghai Yiheng Scientific Instrument Co., Ltd., Shanghai, China).
Crude protein	Kjeldahl nitrogen determination method	Auto Kjeldahl apparatus: Hanon K1100 (Jinan Hanon Instruments Co., Ltd., Jinan, China).
Crude lipid	Soxhlet extraction method	Auto fat analyzer: Hanon SOX606 (Jinan Hanon Instruments Co., Ltd., Jinan, China).
Crude ash	Ashing method (Dry ashing), 560 °C, 6 h	Muffle furnace: XL-2A (Hangzhou Zhuochi Instrument Co., Ltd., Hangzhou, China).
TP ^1^	Coomassie Brilliant Blue method (Model A045-2-2)	Assay kits purchased from Jiancheng Bioengineering Institute (Nanjing, China); Spectrophotometer (Thermo Fisher Multiskan GO, Shanghai, China)
CAT ^2^	Visible light method (Model A007-1-1)	
SOD ^3^	WST-1 method (Model A001-3-2)	
T-AOC ^4^	ABTS method (Model A015-2-1)	
GSH ^5^	Microplate method (Model A006-2-1)	
GPx ^6^	Colorimetric method (Model A005-1-2)	
MDA ^7^	TBA method (Model A003-1-2)	
GR ^8^	UV microplate method (Model A062-1-1)	
LZM ^9^	Turbidimetric method (Model A050-1-1)	

Note: ^1^ TP: total protein; ^2^ CAT: catalase; ^3^ SOD: superoxide dismutase; ^4^ T-AOC: total antioxidant capacity; ^5^ GSH: glutathione; ^6^ GPx: glutathione peroxidase; ^7^ MDA: malondialdehyde; ^8^ GR: glutathione reductase; ^9^ LZM: lysozyme.

**Table 3:** Primer sequences for RT-qPCR analysis.

Gene	Forward (5′-3′)	Reverse (5′-3′)	Primer Source
*il-1β* ^1^	TGAGCCTGAGAGTGCAACTG	AAGTAGCCCTCGAACTTGGC	[20]
*tnf-α* ^2^	GCTCTTCTGGCCATTGGACT	CTTCAGCCCTCCACCGAAAT	
*tgf-β* ^3^	CTGGAGTCCCAGCACAAGAG	AAGTCGATGTAGAGCGAGCG	
*ifn-γ* ^4^	GAACCGCTTGGTCATCTGGA	CCGACTCCTGTGCATCTGTT	
*perk* ^5^	ACGAGAGAGAGCCCACTAGG	TCCTGGGGGAAGACTGAACT	[21]
*atf4* ^6^	TTCCATGACGATCCCAAGCC	CCTCCAGCGTCAGCAGATAC	
*chop* ^7^	AAGATGGCACAGAAGTCGCA	GGCAGATTTGTTGTCCTCCG	
*cflar* ^8^	GTTCGTCTGTTGCCTCCTGA	CTATCTGCGAGGACCAACCC	
*bcl-2* ^9^	ATCTCACATCCACCACTGCG	TGTTGACGCACTCTACGCAT	
*bax* ^10^	TGACATACTGAAGGCGGGAC	TCAGATGTAACGGGGCTGTG	
*tradd* ^11^	TGCATCCAGGCTTCTCGTTC	TCACGAAGTCTCATGGGCTG	
*apaf1* ^12^	CTACTACGCTCACGGCCAAA	TGTACGCTCGGTTGTCCTTC	
*casp9* ^13^	ACGACACGAGTTGTCAGCAT	TCCACACCATACACTGCACC	
*casp3* ^14^	TCATTCGTGTGTGTGCTGCT	CTGTCATGCTCGATGCCACT	
*β-actin* ^15^	AACGGATCCGGTATGTGCAAAGC	GGGTCAGGATACCTCTCTTGCTCTG	

Note: ^1^ *il-1β*: interleukin-1 beta; ^2^ *tnf-α*: tumor necrosis factor-alpha; ^3^ *tgf-β*: transforming growth factor-beta; ^4^ *ifn-γ*: interferon-gamma; ^5^ *perk*: Protein-kinase like endoplasmic reticulum kinase; ^6^ *atf4*: Activating transcription factor 4; ^7^ *chop*: C/EBP homologous protein; ^8^ *cflar*: Cellular FLICE-like inhibitory protein; ^9^ *bcl-2*: B-cell leukemia/lymphoma 2; ^10^ *tradd*: TNF receptor-associated death domain; ^11^ *bax*: Bcl-2-associated X protein; ^12^ *apaf1*: Apoptotic peptidase-activating factor 1; ^13^ *casp9*: Caspase-9; ^14^ *casp3*: Caspase-3; ^15^ *β-actin*: beta-actin.

**Table 4:** Effects of dietary protein level on growth and feed utilization of *C. nasus*.

Protein Level (%)		Items					
		IBW ^1^	FBW ^2^	WGR ^3^	SGR ^4^	FCR ^5^	SR ^6^
35.42		0.87 ± 0.01	3.76 ± 0.07 ^a^	330.51 ± 3.86 ^a^	2.61 ± 0.02 ^a^	4.36 ± 0.25 ^b^	63.5 ± 5.38
39.16		0.87 ± 0.01	3.85 ± 0.05 ^ab^	340.39 ± 6.93 ^a^	2.65 ± 0.03 ^a^	3.74 ± 0.20 ^b^	68.83 ± 2.50
42.96		0.87 ± 0.01	4.08 ± 0.11 ^abc^	367.21 ± 13.48 ^b^	2.75 ± 0.05 ^b^	3.47 ± 0.07 ^a^	78.83 ± 4.06
46.83		0.86 ± 0.01	4.12 ± 0.10 ^c^	376.22 ± 10.36 ^b^	2.79 ± 0.04 ^b^	3.33 ± 0.15 ^a^	75.58 ± 2.27
50.65		0.88 ± 0.01	3.86 ± 0.09 ^ab^	339.47 ± 10.42 ^a^	2.64 ± 0.04 ^a^	3.45 ± 0.11 ^b^	76 ± 1.56
Linear	R^2^	<0.001	0.170	0.153	0.155	0.637	0.511
	*p*-value	0.874	0.127	0.150	0.147	<0.001	0.003
Quadratic	R^2^	0.081	0.627	0.631	0.639	0.878	0.710
	*p*-value	0.604	0.030	0.030	0.020	<0.001	0.004

Note: Data are presented as mean value ± SD (*n* = 3). On the same column, different superscripted letters indicated significances (*p* < 0.05). ^1^ IBW: initial body weight (g); ^2^ FBW: final body weight (g); ^3^ WGR: weight gain rate (%) = 100 × [(FBW (g) − IBW (g))/IBW (g)]; ^4^ SGR: specific growth rate (%/d) = 100 × [(Ln (FBW (g)) − Ln (IBW (g)))/days]; ^5^ FCR: feed conversion ratio = dry feed fed (g)/(FBW (g) − IBW (g)); ^6^ SR: survival rate (%) = 100 × (survival fish number/total fish).

**Table 5:** Effects of dietary protein level on whole body composition of *C. nasus*.

Protein Level (%)	Items			
	Moisture (%)	Crude Protein (%)	Crude Lipid (%)	Crude Ash (%)
35.42	73.69 ± 0.27	16.82 ± 0.63 ^a^	6.25 ± 0.58 ^ab^	2.91 ± 0.24
39.16	73.10 ± 0.25	17.39 ± 0.53 ^ab^	6.21 ± 0.50 ^ab^	2.96 ± 0.32
42.96	73.11 ± 0.07	17.63 ± 0.30 ^b^	6.49 ± 0.35 ^b^	2.77 ± 0.15
46.83	73.10 ± 0.27	17.83 ± 0.59 ^b^	5.80 ± 0.34 ^a^	2.98 ± 0.41
50.65	72.25 ± 0.27	17.59 ± 0.49 ^b^	5.97 ± 0.34 ^ab^	2.90 ± 0.15

Note: Data are presented as mean value ± SD (*n* = 3). Different superscripted letters in the same column indicated a significant difference (*p* < 0.05).

## Data Availability

Data are contained within the article.
